# Stock Market Index Data and indicators for Day Trading as a Binary Classification problem

**DOI:** 10.1016/j.dib.2016.12.044

**Published:** 2016-12-29

**Authors:** Renato Bruni

**Affiliations:** Dip. di Ingegneria Informatica, Automatica e Gestionale, Sapienza Università di Roma, Rome, Italy

## Abstract

Classification is the attribution of labels to records according to a criterion automatically learned from a training set of labeled records. This task is needed in a huge number of practical applications, and consequently it has been studied intensively and several classification algorithms are available today. In finance, a stock market index is a measurement of value of a section of the stock market. It is often used to describe the aggregate trend of a market. One basic financial issue would be forecasting this trend. Clearly, such a stochastic value is very difficult to predict. However, technical analysis is a security analysis methodology developed to forecast the direction of prices through the study of past market data. Day trading consists in buying and selling financial instruments within the same trading day. In this case, one interesting problem is the automatic individuation of favorable days for trading. We model this problem as a binary classification problem, and we provide datasets containing daily index values, the corresponding values of a selection of technical indicators, and the class label, which is 1 if the subsequent time period is favorable for day trading and 0 otherwise. These datasets can be used to test the behavior of different approaches in solving the day trading problem.

**Specifications Table**

**Table:** 

Subject area	*Economics and Finance*
More specific subject area	*Financial Timing, Trading, Stock Index Analysis*
Type of data	Daily time series for two major indices belonging to two different stock markets
How data was acquired	http://www.investing.com/
Data format	*CSV format, can be opened as excel or text*
Experimental factors	*When necessary, data are filtered to remove missing or inaccurate data*
Experimental features	*The data provided consist of some series of daily prices (opening, closing, maximum, minimum), several series of technical indicators, and a class label. They can be used to apply day trading algorithms or classification algorithms*
Data accessibility	*Data is within this article*

**Value of the data**

• The datasets are taken from real-world major financial markets and they are very recent: they range from 20th April 2010 to 12th July 2016.
• The datasets contain a vast selection of financial indicators regarded as highly trend indicative by technical analysis.
• The datasets are filtered and cleaned to remove data errors and missing.
• These datasets can be used as benchmarks by researchers willing to test trading algorithms on real-world recent data.
• These datasets can also be used as benchmarks to test classification strategies on publicly available difficult data.

## 1 Data

We provide daily time series for two major indices belonging to two different stock markets. The first one is the Standard & Poor׳s 500 (S&P 500), which is an American stock market index based on the market capitalizations of 500 large companies having common stock listed on the NYSE or NASDAQ. This is one of the most commonly followed equity indices, and many consider it one of the best representations of the U.S. stock market. The second is the Financial Times Stock Exchange Milano Indice di Borsa (FTSE MIB), which is the primary benchmark Index for the Italian equity markets. It consists of the 40 most-traded stock classes on the exchange, and captures approximately 80% of the domestic market capitalization. For these indices, for each trading day ranging from 20th April 2010 to 12th July 2016, we provide the opening price, closing price, maximum, minimum, and a number of indicators regarded as highly trend indicative by technical analysis (see, e.g., [1], [2], [3] and references therein), as described in more detail in the next section. Each data record corresponds to one day.

We also provide a binary classification for each day: the class is 1 if the subsequent time period is favorable for day trading and 0 otherwise. Data are filtered to check and to correct missing or inaccurate data. Indicators which are computed using the n past observations are available only from the (n+1)-th record of the dataset. The class is not available for the last record. These missing data are encoded as ‘N’. No other missing data appear in the dataset. Data cleaning is indeed an important issue for similar data (see, e.g., [4] for references on this widespread problem). The data provided can be used to test the effectiveness of technical analysis in predicting the trend, or to test the accuracy of classification algorithms.

## 2 Experimental design, materials and methods

Each data record refers to one single trading day. Such a time period is indicated by a subscript t∈{1,…,m}. Each data record is identified with the date and it contains the following values:

• $o_{t}$ denotes the opening price of the index;
• $c_{t}$ denotes its closing price;
• $\max_{t}$ denotes its maximum price;
• $\min_{t}$ denotes its minimum price.

By using the above values, for each trading day we compute:

• the return $r_{t}={(c}_{t}-c_{t-1})/c_{t-1}$ of the index;
• the percentage variation of the closing price $\delta_{t}={100(c}_{t}-c_{t-1})/c_{t-1}$.

After this, we compute the indicators described below. For each of them, the current value is denoted by a subscript t, the previous by t−1, etc.

## 2.1 Momentum

Momentum is conventionally regarded as the basic trend-following indicator. It shows trend by remaining positive while an uptrend is sustained, or negative while a downtrend is sustained. The momentum $M_{t}\left(n\right)$ of the current time period t is computed as the difference between the current closing price $c_{t}$ and the closing price of n days ago $c_{t-n}$

$$M_{t}(n)=\;c_{t}-\;c_{t-n}$$

In our case we use n=5.

Range: momentum can take any real value, either positive or negative. Positive values of momentum denote that the index trend is increasing, and vice versa.

## 2.2 EMA

Moving averages are widely used for the analysis of time series. A simple moving average (SMA) is the unweighted mean of the previous n data of the historical price data, most often the closing price. A weighted moving average (WMA) has multiplying factors to give different weights to the different prices. Usually, recent prices receive more importance than older prices. In particular, an exponential moving average (EMA) applies weighting factors which decrease exponentially in the past, however never reaching zero. ${\mathrm{EMA}}_{t}\left(n\right)$ is computed using the current closing price $c_{i}$ and the EMA of the previous day ${\mathrm{EMA}}_{t-1}\left(n\right)$.

$${\mathrm{EMA}}_{t}\left(n\right)=\left(\frac{2}{n+1}\right)c_{t}\;+\left(1-\frac{2}{n+1}\right){\mathrm{EMA}}_{t-1}\left(n\right)=\;\left(\frac{2}{n+1}\right)\left(c_{t}-{\mathrm{EMA}}_{t-1}\left(n\right)\right)\;+\;{\mathrm{EMA}}_{t-1}\left(n\right)$$

In our case we use n=12 and also n=26.

Range: EMA has the same range of the price of the asset; in general it can take any real positive value.

## 2.3 MACD

Moving Average Convergence/Divergence (MACD) is an oscillator that should reveal changes in the strength, direction, momentum, and duration of a trend in a stock׳s price. The simplest version of MACD is the difference between two moving averages, one over a shorter period n and one over a longer period m.

$${\mathrm{MACD}}_{t}\left(n,m\right)={\mathrm{EMA}}_{t}\left(n\right)-\;{\mathrm{EMA}}_{t}\left(m\right)$$

Further insight can be obtained by using a third moving average of the MACD(n,m) itself over a period s, called "signal line" SL(s). When MACD(n,m) increases and crosses the signal line, it is a bullish signal; when it decreases and crosses the signal line, it is a bearish signal.

$${\mathrm{MACD}}_{t}\left(n,m,s\right)=({\mathrm{EMA}}_{t}\left(n\right)-\;{\mathrm{EMA}}_{t}\left(m\right))-{\mathrm{SL}}_{t}\left(s\right)$$

In our case we use n =12, m=26 and s=9.

Range: MACD can take any real value, either positive or negative. Positive values denotes that the index trend is increasing, and vice versa.

## 2.4 ROI

Return on Investment (ROI) is one way of considering profits in relation to capital invested. Usually it is the ratio between return and invested capital. In our case, we use the average return over the last n days, denoted by $\mathrm{aver}\left\{r_{t},\;r_{t-1},\ldots ,r_{t-n+1}\right\}$, and the current closing value.

$${\mathrm{ROI}}_{t}\left(n\right)=\frac{\mathrm{aver}\left\{r_{t},\;r_{t-1},\ldots ,r_{t-n+1}\right\}}{c_{t}}$$

In our case we use n=10, 20 and 30.

Range: ROI can take any real value, either positive or negative. Positive values denote income, negative ones denote loss.

## 2.5 RSI

Relative Strength index (RSI) is a momentum oscillator that compares the magnitude of recent gains and losses over a specified time period to measure speed and change of price movements of a security. By defining the upward change as $u_{t}=c_{t}-c_{t-1}$ if $c_{t}>\;c_{t-1}$ and 0 otherwise, and the downward change as $d_{t}=c_{t-1}-c_{t}$ if $c_{t}<\;c_{t-1}$ and 0 otherwise, the relative strength RS(n) is the average of the last *n* upward changes divided the average of the last *n* downward changes.

$${\mathrm{RS}}_{t}\left(n\right)=\;\frac{\mathrm{aver}\left\{u_{t},u_{t-1},\ldots ,u_{t-n+1}\right\}}{\mathrm{aver}\left\{d_{t},d_{t-1},\ldots ,d_{t-n+1}\right\}}$$

Then, RSI is computed as follows

$${\mathrm{RSI}}_{t}\left(n\right)=100-\;\frac{100}{{1+\mathrm{RS}}_{t}}$$

RSI is considered a signal of overbought when above 70 and a signal of oversold when below 30.

In our case we use n=10, 14 and 30.

Range: RSI oscillates between 0 and 100. It is near to 0 when the corresponding upward changes are near to 0, it is near to 100 when the corresponding downward changes are near to 0.

## 2.6 STOCHRSI

Stochastic oscillators attempt to predict price turning points by comparing the closing price of a security to its price range. This concept can be applied to the RSI itself, obtaining the Stochastic RSI (SRSI). By computing the RSI range from its minimum in the last *n* periods $\min\left\{{\mathrm{RSI}}_{t},{\mathrm{RSI}}_{t-1},\ldots ,{\mathrm{RSI}}_{t-n+1}\right\}$ and its maximum in the last *n* periods $\max\left\{{\mathrm{RSI}}_{t},\;{\mathrm{RSI}}_{t-1},\ldots ,{\mathrm{RSI}}_{t-n+1}\right\}$, the SRSI is defined as follows.

$${\mathrm{SRSI}}_{t}\left(n\right)=\;\frac{{\mathrm{RSI}}_{t}\left(n\right)-\min\left\{{\mathrm{RSI}}_{t},{\mathrm{RSI}}_{t-1},\ldots ,{\mathrm{RSI}}_{t-n+1}\right\}}{\max\left\{{\mathrm{RSI}}_{t},{\mathrm{RSI}}_{t-1},\ldots ,{\mathrm{RSI}}_{t-n+1}\right\}-\min\left\{{\mathrm{RSI}}_{t},{\mathrm{RSI}}_{t-1},\ldots ,{\mathrm{RSI}}_{t-n+1}\right\}}$$

In our case we use *n* = 10, 14 and 30.

Range: its range is between 0 and 1.

## 2.7 ATR

Average True Range (ATR) measures the degree of price volatility. The rage of a price is simply defined as $\max_{t}-\min_{t}$ , the True Range (TR) extends it to yesterday׳s closing price if it was outside of today׳s range:

$${\mathrm{TR}}_{t}=\max\{\max_{t},\;c_{t-1}\}-\min\{\min_{t},\;c_{t-1}\}$$

Now, by denoting with ${\mathrm{EMA}}_{t}\left(n,X\right)$ the exponential moving average of a generic X over the last *n* periods, we have that ATR is the exponential moving average of the TR:

$${\mathrm{ATR}}_{t}\left(n\right)=\;{\mathrm{EMA}}_{t}\left(n,\mathrm{TR}\right)$$

In our case we use *n*=14.

Range: it is any positive value.

## 2.8 ADX

Average Directional Index (ADX) does not indicate trend direction or momentum, only trend strength. It is computed using the positive directional indicator (+DI), the negative directional indicator (-DI), and the Average True Range (ATR).

By defining the upmove as ${\mathrm{up}}_{t}=\max_{t}-\max_{t-1}$ and the downmove as ${\mathrm{dw}}_{t}=\min_{t-1}-\min_{t}$ ,

$$\mathrm{if}\;{\mathrm{up}}_{t}>{\mathrm{dw}}_{t}\;\mathrm{and}\;{\mathrm{up}}_{t}>\;0\;\mathrm{then}{+\mathrm{DM}}_{t}={\mathrm{up}}_{t},\;\mathrm{otherwise}\;{+\mathrm{DM}}_{t}=0;\mathrm{if}\;{\mathrm{dw}}_{t}>{\mathrm{up}}_{t}\;\mathrm{and}\;{\mathrm{dw}}_{t}>\;0\;\mathrm{then}{-\mathrm{DM}}_{t}={\mathrm{dw}}_{t},\;\mathrm{otherwise}{-\mathrm{DM}}_{t}=0.\;$$

Now, recalling that ${\mathrm{EMA}}_{t}\left(n,X\right)$ denotes the exponential moving average of X over the last *n* periods, we compute

$${+\mathrm{DI}}_{t}\left(n\right)=\frac{100\;{\mathrm{EMA}}_{t}(n,+\mathrm{DM})}{{\mathrm{ATR}}_{t}(n)}{-\mathrm{DI}}_{t}\left(n\right)=\frac{100\;{\mathrm{EMA}}_{t}(n,-\mathrm{DM})}{{\mathrm{ATR}}_{t}(n)}$$

ADX is finally computed as follows, with abs(.) denoting the absolute value:

$${\mathrm{ADX}}_{t}\left(n\right)=100\;\frac{{\mathrm{EMA}}_{t}(n,\mathrm{abs}\left(+\mathrm{DI}-\left(-\mathrm{DI}\right)\right))}{+\mathrm{DI}+\left(-\mathrm{DI}\right)}$$

In our case we use n=14.

Range: it ranges between 0 and 100. Generally, ADX values below 20 indicate trend weakness, and values above 40 indicate trend strength.

## 2.9 Williams %R

Williams %R is an oscillator that analyzes whether a stock or commodity market is trading near the high or the low, or somewhere in between, of its recent trading range.

$${\%R}_{t}\left(n\right)=-100\;\frac{{\max\left\{\max_{t},\max_{t-1},\ldots ,\max_{t-n+1}\right\}-\;c}_{t}}{\max\left\{\max_{t},\max_{t-1},\ldots ,\max_{t-n+1}\right\}-\min\left\{\min_{t},\min_{t-1},\ldots ,\min_{t-n+1}\right\}}$$

In our case we use *n*=14.

Range: it ranges between −100 and 0. A value of −100 means that the close today was the lowest low of the past *n* days, and 0 means that today׳s close was the highest high of the past *n* days.

## 2.10 CCI

Commodity Channel Index (CCI) is used to identify cyclical trends not only in commodities, but also equities and currencies. Define the Typical Price (TP) as follows.

$${\mathrm{TP}}_{t}\;=\frac{\max_{t}+\min_{t}+c_{t}}{3}$$

By computing the simple average over the last *n* periods of the typical price and its standard deviation, CCI is defined as follows.

$${\mathrm{CCI}}_{t}\left(n\right)=\frac{{\mathrm{TP}}_{t}-\mathrm{aver}\left\{{\mathrm{TP}}_{t},{\mathrm{TP}}_{t-1},\ldots ,{\mathrm{TP}}_{t-n+1}\right\}}{0.015\mathrm{dev}\left\{{\mathrm{TP}}_{t},{\mathrm{TP}}_{t-1},\ldots ,{\mathrm{TP}}_{t-n+1}\right\}}$$

In our case we use n=20.

Range: the CCI fluctuates above and below zero. The constant 0.015 should ensure that approximately 70−80% of CCI values lay between −100 and +100.

## 2.11 UO

Ultimate Oscillator (UO) uses buying or selling "pressure", represented by where the daily closing price falls within the daily true range. The Buying Pressure (BP) and the True Range (TR) are computed as follows.

$${\mathrm{BP}}_{t}=\;c_{t}-\min\{\min_{t},\;c_{t-1}\}\;{\mathrm{TR}}_{t}=\max\{\max_{t},\;\;c_{t-1}\}-\min\{\min_{t},\;c_{t-1}\}$$

Then, the total buying pressure over the past n days is computed as follows.

$${\mathrm{AVG}}_{t}\left(n\right)=\frac{{\mathrm{BP}}_{t}+\;{\mathrm{BP}}_{t-1}+{\mathrm{BP}}_{t-n+1}}{{\mathrm{TR}}_{t}+\;{\mathrm{TR}}_{t-1}+{\mathrm{TR}}_{t-n+1}}$$

Such a total buying pressure is computed for short, intermediate and long time intervals, and the UO is:

$${\mathrm{UO}}_{t}\left(n,m,s\right)=100\;\frac{4\;{\mathrm{AVG}}_{t}\left(n\right)+2\;{\mathrm{AVG}}_{t}\left(m\right)+{\mathrm{AVG}}_{t}\left(s\right)}{7}$$

In our case we use n=7, m=14 and s=28.

Range: it ranges between 0 and 100.

## 2.12 Class

The problem of data classification is the attribution of labels to records according to a criterion automatically learned from a training set, that is a set of records that already have a class. Classification is a very important data mining task (see also [5]), and many classifications algorithms are today available (e.g., [6]). We assign the class to each record, so that any portion of the dataset can be used as training set. After this learning phase, the classification algorithm will be able to predict the class for the rest of the records. The accuracy of such a prediction can be computed by comparing it with the real class, which is the one given in the dataset.

The class that we assign to each daily record is 1 if the subsequent day is favorable for intra-day trading and 0 otherwise. Favorable for intra-day trading means that the increase between the opening price and the closing price of the same day is large enough for obtaining a profit by buying at the opening price and selling at the closing price. A threshold must be selected to define “large enough”; we select the value 0.3%, which should provide a reasonable opportunity for profit. Therefore, the class is defined as follows. Its prediction would allow to perform intra-day trading in the following day, as described above, or it could possibly be used to define inter-day trading strategies.

$$Class_{t}=\{\begin{array}{cc} 1 & \mathrm{if}\;100\;(c_{t+1\;}-o_{t+1})/o_{t+1}\geq 0.3\\ 0 & \mathrm{otherwise} \end{array}$$

Note that the class of a given day is clearly not computable from the data available up to that day. However, we assigned it for the whole dataset by simply looking, for each day, at the following day.

According to technical analysis, there should be some kind of relation between the above described indicators at day t and the market evolution at day t+1, that determines the class of day t (see for example [7]). The classification algorithm aims at discovering such a relation by predicting the class using the above described indicators. For an analysis of the existence of profit opportunities with respect to the market index, see also [8].

The datasets are provided in CSV format, that can be opened with MS Excel or as text file.
